# Importance of Non-Selective Cation Channel TRPV4 Interaction with Cytoskeleton and Their Reciprocal Regulations in Cultured Cells

**DOI:** 10.1371/journal.pone.0011654

**Published:** 2010-07-19

**Authors:** Chandan Goswami, Julia Kuhn, Paul A. Heppenstall, Tim Hucho

**Affiliations:** 1 Signal Transduction in Pain and Mental Retardation, Department for Molecular Human Genetics Max Planck Institute for Molecular Genetics, Berlin, Germany; 2 National Institute of Science Education and Research, Bhubaneswar, India; 3 Klinik für Anaesthesiologie und Operative Intensivmedizin, Charité Universitätsmedizin Berlin, Campus Benjamin Franklin, Berlin, Germany; Duke University, United States of America

## Abstract

**Background:**

TRPV4 and the cellular cytoskeleton have each been reported to influence cellular mechanosensitive processes as well as the development of mechanical hyperalgesia. If and how TRPV4 interacts with the microtubule and actin cytoskeleton at a molecular and functional level is not known.

**Methodology and Principal Findings:**

We investigated the interaction of TRPV4 with cytoskeletal components biochemically, cell biologically by observing morphological changes of DRG-neurons and DRG-neuron-derived F-11 cells, as well as functionally with calcium imaging. We find that TRPV4 physically interacts with tubulin, actin and neurofilament proteins as well as the nociceptive molecules PKCε and CamKII. The C-terminus of TRPV4 is sufficient for the direct interaction with tubulin and actin, both with their soluble and their polymeric forms. Actin and tubulin compete for binding. The interaction with TRPV4 stabilizes microtubules even under depolymerizing conditions *in vitro*. Accordingly, in cellular systems TRPV4 colocalizes with actin and microtubules enriched structures at submembranous regions. Both expression and activation of TRPV4 induces striking morphological changes affecting lamellipodial, filopodial, growth cone, and neurite structures in non-neuronal cells, in DRG-neuron derived F11 cells, and also in IB4-positive DRG neurons. The functional interaction of TRPV4 and the cytoskeleton is mutual as Taxol, a microtubule stabilizer, reduces the Ca^2+^-influx via TRPV4.

**Conclusions and Significance:**

TRPV4 acts as a regulator for both, the microtubule and the actin. In turn, we describe that microtubule dynamics are an important regulator of TRPV4 activity. TRPV4 forms a supra-molecular complex containing cytoskeletal proteins and regulatory kinases. Thereby it can integrate signaling of various intracellular second messengers and signaling cascades, as well as cytoskeletal dynamics. This study points out the existence of cross-talks between non-selective cation channels and cytoskeleton at multiple levels. These cross talks may help us to understand the molecular basis of the Taxol-induced neuropathic pain development commonly observed in cancer patients.

## Introduction

Transient receptor potential vanilloid sub type 4 (TRPV4) is a member of TRP super family of Ca^2+^-permeable non-selective cation channels. This polymodal receptor is involved in cellular processes such as mechanosensation, osmosensation and thermosensation [1]–[5]. Some of these sensory functions are well conserved in different species. For example, mammalian TRPV4 can rescue mechanosensitive defects observed in *C.elegans* OSM-9 mutants [5]. In higher organisms TRPV4 is endogenously expressed in nociceptive dorsal root ganglion (DRG) neurons but also in many non-neuronal tissues and cells such as skin, kidney corneal epithelial cells [6], cerebral microvascular endothelial cells [7], cortical astrocytes [8], tracheal epithelial cells [9], keratinocyte cell lines [10] and in other cells. The widespread distribution of TRPV4 is indicative of its involvement in various physiological functions. Indeed, TRPV4 is of importance in shear stress-induced vasodilation [11] as well as in auditory functions [12]–[13]. Recently TRPV4 gained importance as it has been linked with the development of different pathophysiological conditions such as neuropathic pain, cystic fibrosis, brachyolmia and cancer [14]–[18].

From several reports, the involvement of cytoskeleton can be correlated with the localization and function of TRPV4. For example, TRPV4 is found in structures like cilia in various tissues and cells [9], [19]–[21] and in lamellipodia, where it regulates the dynamics of cytoskeleton [22]–[23]. Many cellular functions involving TRPV4 are known to require active participation of the cytoskeleton. For example, TRPV4 activity is central to cytoskeleton-dependent/mediated regulatory volume decrease of cells [6], [10], [24], a process where actin-binding proteins contribute to cell volume regulatory ion channel activation [24]–[26]. In addition, TRPV4 has a conserved role in mechanotransduction, a complex process that involves both actin and microtubule cytoskeletal components [27]–[29]. The interplay of TRPV4 with microtubule cytoskeleton also appears on a behavioural level, where alteration of microtubule dynamics by Taxol induces a TRPV4-dependent painful peripheral neuropathy [30]. While all these cellular and behavioural studies strongly suggest that TRPV4 shares a functional relation with the cytoskeleton, so far a direct link of TRPV4 with the cytoskeleton has not been demonstrated. Thus, a molecular mechanism for the role of TRPV4 and the cytoskeleton in pain, mechanosensation as well as other cellular functions remains elusive.

Recently, we have established a functional interplay between TRPV1, a close homologue of TRPV4, and the microtubule cytoskeleton [31]–[35]. We demonstrated the physical interaction of microtubule cytoskeleton with TRPV1 via two novel tubulin-binding motifs [36]–[37]. Based on our previous experiments done on TRPV1 and the sequence homology between TRPV1 and TRPV4, we predicted that TRPV4 might interact with tubulin via its C-terminal domain. Therefore, in this work we set out to explore if TRPV4 physically and functionally interacts with actin and microtubule cytoskeletal components.

## Results

### TRPV4 interacts with endogenous actin and tubulin

**Figure 1:**
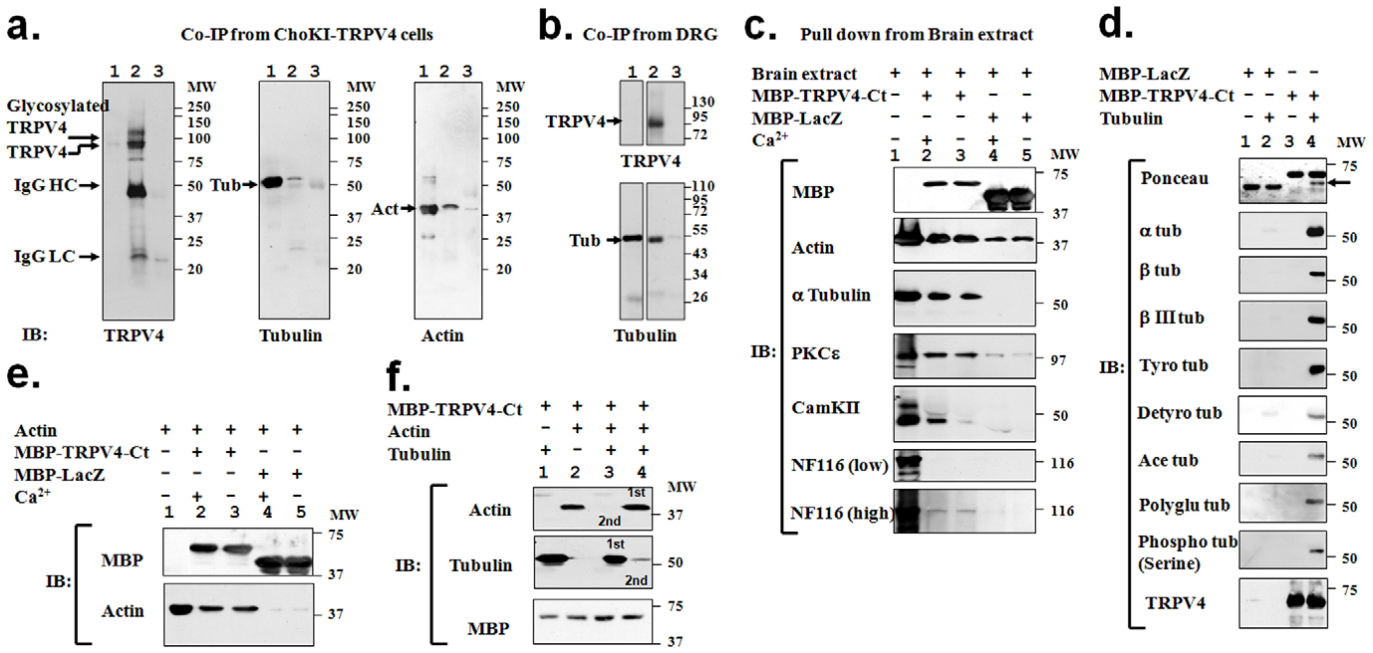
Interaction of soluble tubulin and actin with TRPV4. **a.** Co-immunoprecipitation of actin and tubulin with TRPV4. Cell extracts from CHO-KI cells stably expressing TRPV4 (lane 1) was immunoprecipitated by TRPV4 antibody (lane 2) or by a non-specific antibody (lane 3). Blots were probed for TRPV4 (left side), tubulin (middle) and actin (right side). **b.** Co-immunoprecipitation of tubulin with TRPV4. Extracts from DRG (lane 1) was immunoprecipitated by TRPV4 antibody (lane 2) or by a non-specific antibody (lane 3). Blots were probed for TRPV4 (upper panel) and tubulin (lower side). **c.** MBP-TRPV4-Ct (lane 2-3) but not MBP-LacZ (lane 4–5) forms specific complexes when incubated with mammalian brain extract (lane 1), both in presence (lane 2 and 4) or absence (lane 3 and 5) of Ca^2+^ (1 mM). Presence of PKCε, actin and tubulin are observed only in lane 2 and 3. Neurofilament in the pull down samples is visible only after exposing for a prolonged time. Presence of CamKII is noted only in the presence of Ca^2+^ (lane 2). Note that the amount of MBP-LacZ used, as a negative control for the pull down experiment is much more than MBP-TRPV4-Ct. **d.** Tubulin interacts with TRPV4-Ct directly. MBP-LacZ (lane 1–2) or MBP-TRPV4-Ct (lane 3–4) was incubated with buffer only (lane 1 and 3) or with purified tubulin (lane 2 and 4). Pulled down samples were probed for different isotype-specific and different post-translationally modified tubulins. **e.** Actin interacts directly with TRPV4-Ct. MBP-TRPV4-Ct (lane 1–2) or MBP-LacZ (lane 3–4) was incubated with purified actin (lane 1–4) either in the presence (lane 1-and 3) or absence (lane 2 and 4) of Ca^2+^ (1 mM) and subsequently probed for bound actin. **f.** Soluble tubulin and actin competes for the C-terminal cytoplasmic fragment of TRPV4. MBP-TRPV4-Ct was incubated with only tubulin (lane 1), with only actin (lane 2), or both tubulin and actin in a sequential manner (lane 3–4). Prior incubation of tubulin inhibits further binding of actin (lane 3). Similarly, prior incubation of actin significantly reduces the further binding of tubulin (lane 4).

In order to test if TRPV4 interacts with cytoskeletal proteins like tubulin and actin, we performed co-immunoprecipitation experiments with affinity purified TRPV4 antibodies. CHO-KI-TRPV4 stable cell lines were used, which express low levels of TRPV4. In immunoblot analysis, we observed that TRPV4 antibodies precipitated TRPV4 together with actin and tubulin proteins (Fig. 1a). Presence of tubulin and actin was not observed when a similar co-immunoprecipitation was performed from the same cell extract using an antibody, which was not raised against TRPV4. To confirm further that the tubulin interaction is occurring even in endogenous tissues, we isolate DRG neurons from rat and performed similar co-immunoprecipitation experiments with affinity purified TRPV4 antibodies. We observed that tubulin co-immunoprecipitated with TRPV4 even from DRG neurons (1b).

### The C-terminus of TRPV4 is sufficient for interaction with actin and tubulin

To identify, which part of the TRPV4 interacts with actin and/or tubulin proteins, we performed a pull down experiment using maltose-binding-protein (MBP)-fused to the N- and C-termini of TRPV4. According to our prediction [37], at least one tubulin-binding site is located within the C-terminus of TRPV4 (Fig. S1) and as the expression of the N-terminal cytoplasmic fragment remained poor, we restricted our study to the C-terminus of TRPV4 only. Pull down was performed from adult porcine brain homogenate as well as from F11 cell lysate, a fusion-cell of rat DRG neurons and mouse neuroblastoma cells [38]. In immunoblot analysis, we observed MBP-TRPV4-C-terminus fusion protein (MBP-TRPV4-Ct) but not the control fusion protein MBP-LacZ to pull down soluble tubulin and actin (Fig. 1c, S2). When probed for the presence of another cytoskeletal element, namely soluble neurofilament proteins, only minimal amounts of NF116 kDa and no NF200 kDa were detected.

As activation of TRPV4 results in high Ca^2+^-influx, we tested if higher concentration of Ca^2+^ could modulate these interactions. However, we observed that tubulin and actin interaction with TRPV4 do not depend on Ca^2+^ (Fig. 1c, S2).

### TRPV4-Ct forms a Ca^2+^-sensitive supra-molecular signaling complex made of actin, tubulin as well as nociceptive signaling components PKCε and CamKII

Previously we have demonstrated that mechanical hyperalgesia induced agonists of novel estrogen receptor GPR30 strongly correlates with the translocation of PKCε in IB4 (+) neurons [39]. Moreover, mechanical hyperalgesia can be induced by activation of the epsilon isoform of PKC (PKCε), a well-described pro-nociceptive signaling molecule [39]–[43]. In addition, calmodulin, and CamKII function has been linked with the chronic inflammatory pain [44]–[46]. Thus, we tested if the TRPV4-actin/tubulin complex also contains PKCε and/or CamKII. We found CamKII to be present in the eluates of the pull-down material both from soluble brain and F11 cell extract, but only in the presence of Ca^2+^ (Fig. 1c, Fig. S1). In addition, we also detected PKCε in the MBP-TRPV4-Ct complex, both in presence and absence of Ca^2+^. Presence of PKCε or CamKII was not observed with the control protein MBP-LacZ. Thus, using two different biological sources, our results indicate that TRPV4-Ct can form supra-molecular complexes consisting of structural and signaling proteins.

### MBP-TRPV4-Ct interacts directly with soluble tubulin and actin

To test if TRPV4-Ct interacts directly with tubulin and actin, we performed pull down experiments with the purified MBP-TRPV4-Ct, tubulin, and actin. As expected, MBP-TRPV4-Ct but not MBP-LacZ pulls down tubulin (Fig. 1d). Tubulin is subjected to different types of post-translational modification, which regulate the process of microtubule stabilization, destabilization and maturation. Thus, we tested for the presence of various post-translationally modified tubulins in the complex formed with MBP-TRPV4-Ct. Indeed we found a large number of these post-translationally modified tubulins and neuron-specific β-III tubulin (Fig. 1d).

Next we tested if also soluble actin interacts directly with MBP-TRPV4-Ct. In immunoblot analysis, we observed that the MBP-TRPV4-Ct pulls down purified actin, while under the same conditions MBP-LacZ was unable to pull down any actin (Fig. 1e). Again, we observed no influence of Ca^2+^ on this interaction.

### Tubulin and actin compete for binding to MBP-TRPV4-Ct

To understand if tubulin binding affects actin binding and vice versa, we performed a competition experiment between soluble tubulin and soluble actin for the binding to MBP-TRPV4-Ct. Soluble tubulin and actin were added to MBP-TRPV4-Ct sequentially before being analysed for their binding to TRPV4 (Fig. 1f). As control either soluble tubulin or soluble actin were used alone and washed in the same manner. We observed that the amount of bound tubulin was strongly reduced if MBP-TRPV4-Ct was initially incubated with actin (Fig. 1f). Inversely, the amount of bound actin is very low if MBP-TRPV4-Ct is initially incubated with tubulin. These results indicate that both actin and tubulin compete for binding to MBP-TRPV4-Ct.

### MBP-TRPV4-Ct interacts with polymerized actin and tubulin filaments and favours formation of stable microtubules

**Figure 2:**
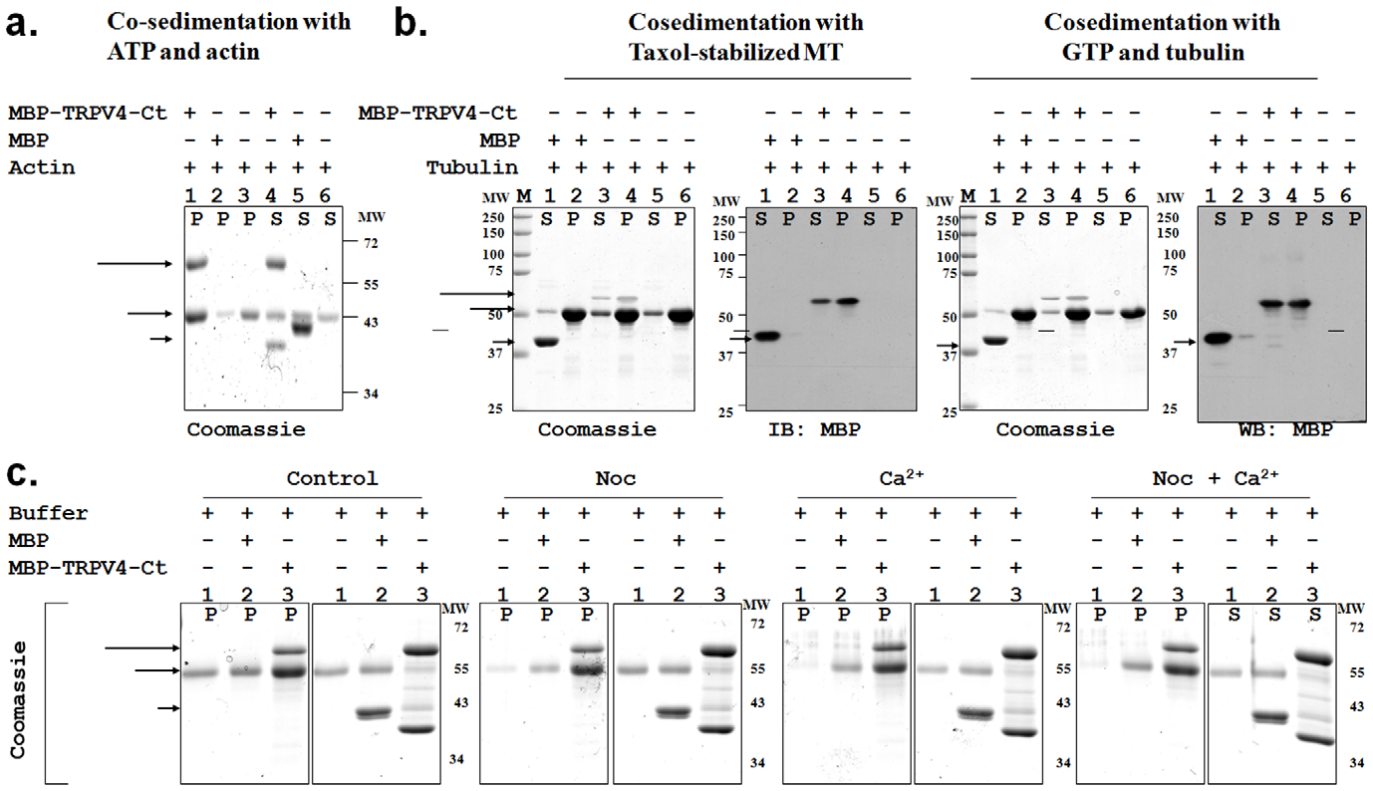
TRPV4 interacts directly with polymerized actin and microtubule filaments. MBP and MBP-TRPV4-Ct were centrifuged at 70000 g/30 min/4°C and only soluble proteins present in the supernatant were used for all co-sedimentation experiments. **a.** MBP-TRPV4-Ct co-sediments with polymerized actin filaments. Actin was polymerized either in presence of MBP-TRPV4-Ct (lane 1 and 4), in presence of MBP only (lane 2 and 5) or in buffer only (lane 3 and 6). Polymerized actin filaments and associated proteins were isolated from remaining soluble actin and unbound proteins by centrifugal separation of pellets (**P**, lane 1–3) from corresponding supernatants (**S**, lane 4–6). The entire amount of MBP remains in the supernatant (lane 5) while a significant amount of MBP-TRPV4-Ct appears in the pellet (lane 1). Arrows indicate the position of respective proteins. **b.** MBP-TRPV4-Ct co-sediments with microtubules. Taxol-stabilized microtubules (left panel) were incubated with MBP (lane 1–2), MBP-TRPV4-Ct (lane 3–4) or with buffer only (lane 5–6) followed by the centrifugal separation of pellet (**P**) consisting MT and bound proteins from supernatant (**S**) consisting of soluble tubulin and other unbound proteins (left side panel). In right side panel, soluble tubulin and GTP was incubated with MBP (lane 1–2), MBP-TRPV4-Ct (lane 3–4) or buffer only (lane 5–6) followed by separation of pellet (**P**) and supernatant (**S**). Note the specific presence of MBP-TRPV4-Ct in the pellet in both cases (in lane 4). **c.** MBP-TRPV4-Ct stabilizes microtubules against depolymerizing factors. Microtubules was formed form soluble tubulin in buffer (lane 1), along with MBP (lane 2) or along with MBP-TRPV4-Ct (lane 3) in control condition (left most panel), in presence of Nocodazole (middle left panel), in presence of Ca^2+^ (middle right side) or in presence of both Nocodazole and Ca^2+^ (right most). Microtubules and bound proteins present in the pellet fraction (**P**) were isolated from unpolymerized tubulin and unbound proteins remaining in the supernatant (**S**) by centrifugal separation. Note the enhancement of polymerized microtubules (represented by tubulin present in lane 3, **P** fraction in every conditions) due to the presence of MBP-TRPV4-Ct.

Next we addressed whether TRPV4 can also bind to polymerized filaments. We observed that MBP-TRPV4-Ct co-sedimented with polymerized actin and thus appeared in the pellet fraction (Fig. 2a). Under the same conditions, MBP only showed no interaction with polymerized actin filaments.

Here we probed if the MBP-TRPV4-Ct can also interact with polymerized microtubules. We observed that MBP-TRPV4-Ct but not MBP alone co-sedimented with Taxol-stabilized polymerized microtubules (Fig. 2b, left side). In addition, we observed that if MBP-TRPV4-Ct was added to saturated tubulin dimer solution during GTP-induced microtubule formation, co-sedimentation of MBP-TRPV4-Ct with polymerized microtubules was also observed (Fig. 2b, right side). Again, only MBP, the control protein failed to bind polymerized microtubules. These results confirm that MBP-TRPV4-Ct directly interacts with filamentous microtubules.

We tested if MBP-TRPV4-Ct can change the physico-chemical properties of the microtubules. For that purpose, we analysed the stability of the microtubules by comparing the amount of microtubules formed under depolymerization conditions, both in the presence or absence of MBP-TRPV4-Ct. We observed that the presence of MBP-TRPV4-Ct favours microtubule formation even in presence of Nocodazole, Ca^2+^, or both (Fig. 2c). In contrast, in absence of MBP-TRPV4-Ct much lower amount of microtubules was formed. MBP as a control protein does not interact with the polymerised microtubules and thus fails to provide stability to the microtubules. This result suggests a stabilization effect of MBP-TRPV4-Ct on microtubules.

### TRPV4 localizes to actin- and microtubule-enriched regions in F11 cell

Based on a RT-PCR analysis demonstrating the amplification of small mRNA fragment, endogenous expression of TRPV4 in F11 cell has been proposed [47]. However, by immunofluorescence analysis two different polyclonal antibodies which recognise the C-terminal region of TRPV4, we could not observe any specific endogenous expression of TRPV4 in F11 cells. To investigate if the biochemical interaction under *in vitro* conditions also occurs *in vivo*, we expressed TRPV4 in F11 cells as well as in other non-neuronal cells and performed co-localization experiments. The purpose of TRPV4 overexpression in F-11 cells is to mimic the physiological situation in DRG neurons.

Using fluorescent labelled Phalloidin, we observed co-localization of TRPV4 with actin at various actin cytoskeleton enriched regions, such as the actin ribs of the cortical membranous regions, and at filopodial and lamellipodial structures (Fig. S3).

**Figure 3:**
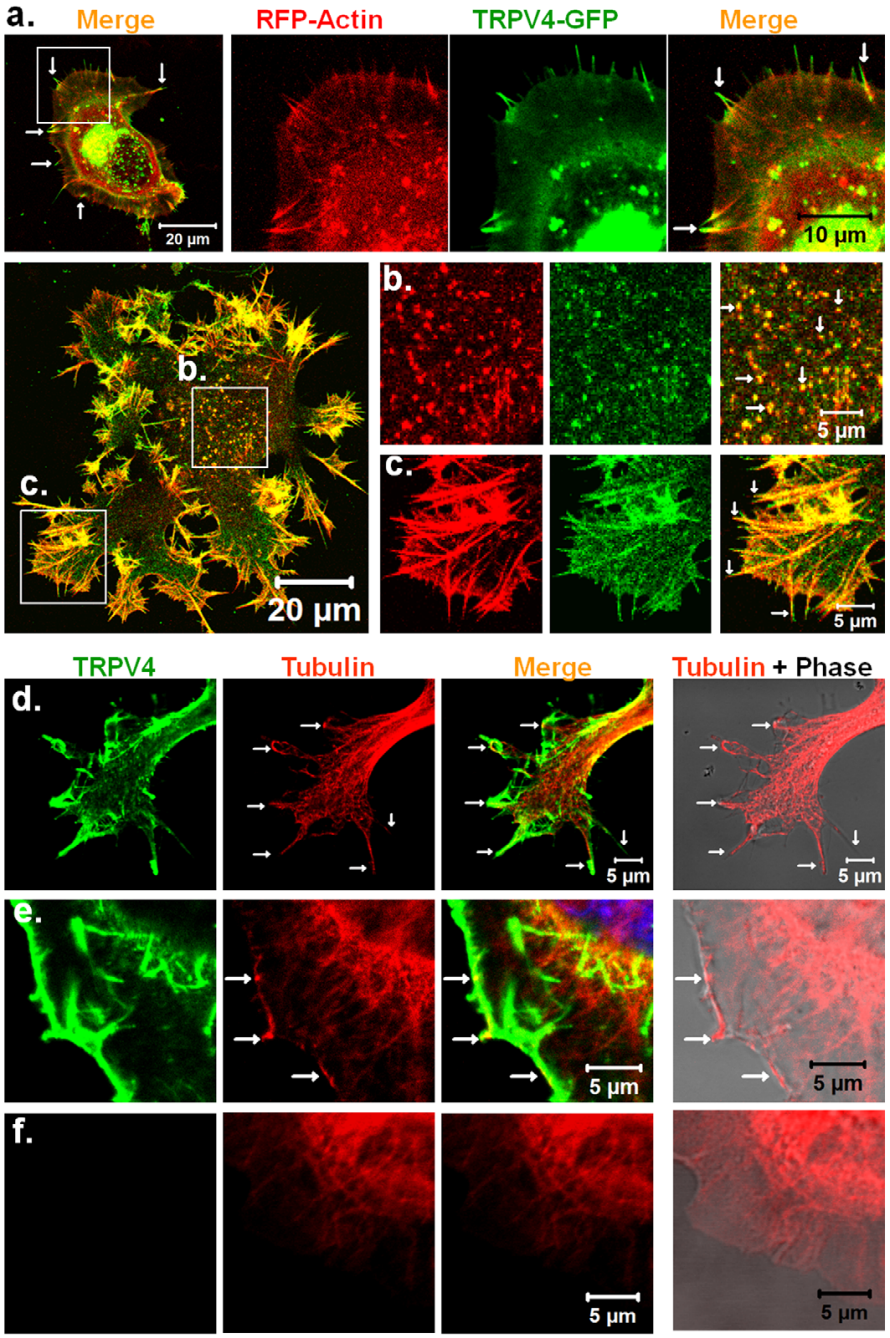
TRPV4 co-localizes with actin and microtubule cytoskeleton. **a–c.** Shown are the live-cell confocal images of F11 cells expressing TRV4-GFP (green) and RFP-actin (red). Presences of TRPV4-GFP specifically in actin-enriched structures are shown. **a.** Enlarged view of lamellipodia and at the tip of the actin filaments are shown. **b–c.** Enlarged view of focal adhesion point-like structures (b) and cell cortex with actin ribs (c) are shown. Arrows indicate the localization of TRPV4-GFP at filopodial tips. **d–f.** TRPV4 co-localizes with microtubule cytoskeleton. Shown are the confocal images of F11 cells immunostained for TRPV4 (green) and tyrosinated tubulin (red). Arrows indicate presence and acumulation of microtubules in thin filopodial structures (d, upper panel) and thin lamellipodial structures (e, middle panel). The status of the microtubules in the non-transfected cells are shown in below (f, lower panel).

As some of these structures are very dynamic and sensitive to environmental changes such as temperature drops or media composition, we attempted to confirm that the observed co-localization in these structures was not a fixation artefact. Thus, we performed live cell imaging by expressing TRPV4-GFP and RFP-actin in F11 cells and we observed co-localization at filopodia, at lamellipodia, and also at cortical actin-rich structures (Fig. 3a-c). We also noted co-localization at actin-rich structures, which resemble focal adhesion points (Fig. 3b). This is in agreement with the fact that focal adhesion points are important for cellular mechanosensory functions [48].

Next, we addressed if TRPV4 similarly co-localizes with microtubules. We observed co-localization between TRPV4 and microtubules in fixed F11 cells all along neurite-like structures. Co-localization was observed at the plasma membrane and at membrane ruffles that are enriched in TRPV4 (Fig. 3d-e). In addition, we noted the presence of numerous microtubule ends at the submembranous regions. Often these microtubule ends extended to the plasma membrane and seemed to be stabilized at the membranous regions containing TRPV4. Co-localization of tubulin and TRPV4 was also observed in filopodial structures that were developed from growth cones, from neurite-like structures as well as from cell bodies. This kind of submembranous tubulin accumulation was not observed in non-transfected cells (Fig. 3f), implying that this effect is primarily due to the presence of TRPV4.

### Activation of TRPV4 results in fast retraction of growth cones in F11 cell

**Figure 4:**
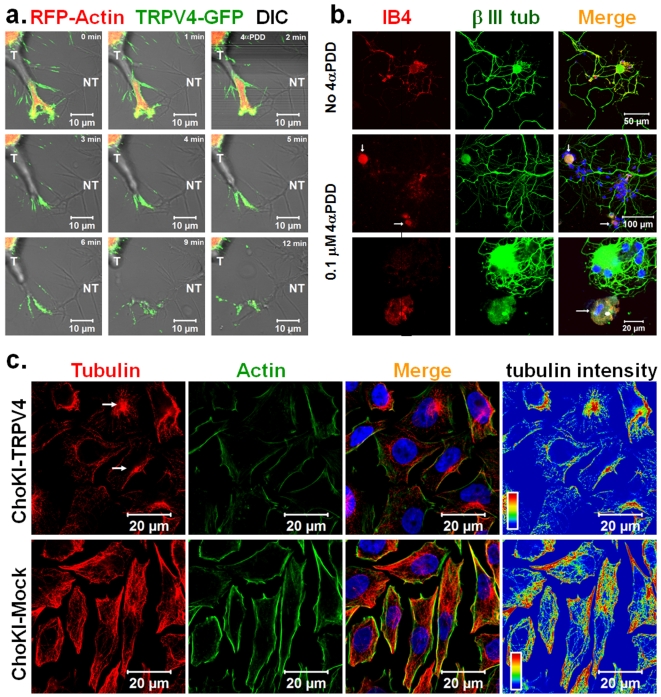
TRPV4 localizes in the growth cone and regulates axonal motility as activation of TRPV4 results in microtubule disassembly. **a**. Shown are the confocal time series images of live F11 cell expressing TRPV4-GFP (green) and RFP-Actin (red). Fluorescence images were superimposed on the DIC images. Addition of 4αPDD (1 µM) results in growth cone retraction of the transfected cell (T) but not from the non-transfected (NT) cells. **b.** Prolonged activation of endogenous TRPV4 reduces neurite outgrowth. Shown are the images of cultured DRG neurons stained for IB4 (red) and βIII tubulin (green). IB4-positive neurons extend their neurites in control condition (upper panel). Majority of the IB4-positive neurons do not produce any neurite when 4αPDD at low dose (0.1 µM) was applied for 36 hours (middle panel). An enlarged view of an IB4-positive and an IB4-negative neuron is shown in the lower panel. Note that the majority of the IB4-negative neurons remain unaffected even in the presence of 4αPDD. **c.** CHO-KI-TRPV4 cells that express low level of TRPV4 or CHO-KI-Mock cells that do not express TRPV4 were activated with 4αPDD (1 µM). After activation, cells were extracted by detergent in isotonic buffer and fixed subsequently by PFA. Cells were immunostained for actin (green) and tubulin (red). CHO-KI-TRPV4 cells loose all the peripheral microtubules but retain filamentous actin after activation and extraction. The stable MTOC regions are marked with arrows. In contrast, CHO-KI-Mock cells remain unaffected. Intensity of the microtubule is shown (red and blue indicate highest and lowest intensity respectively).

TRPV4 localizes to growth cones when expressed in F11 cells. So we tested if TRPV4 activation can alter the morphology and movement of growth cones. For that purpose we expressed TRPV4-GFP along with RFP-actin and performed live cell imaging. We observed that in response to 4α-phorbol-didecanoate (4αPDD, 1 µM), a TRPV4-specific agonist [22], neurites from TRPV4-GFP expressing F11 cells show rapid change in its morphology and induce multiple varicosities (Fig. S4). In response to 4αPDD, TRPV4-GFP containing growth cones retract quickly (Fig. 4a). Under the same conditions growth cones developing from non-transfected cells do not show any retraction, thus assuring specificity of the pharmacological treatment. These results strongly suggest that TRPV4 can regulate the growth cone motility.

### Long-term exposure to a TRPV4 agonist restricts neurite outgrowth in a subset of primary DRG neurons

To validate the effect of TRPV4 activation on growth cones in a system with endogenous TRPV4 expression, we used primary neurons and tested the effect of TRPV4 activation. DRG neurons from adult male rat were cultured for 5 days and treated with 4αPDD (1 µM) for short-term (20 minutes). Live cell-imaging revealed indications of slow retraction of neurites in response to 4αPDD in some neurites, but did not show immediate retraction or varicosity formation (data not shown). Thus we tested if long-term exposure to a TRPV4 agonist but a low-level activation of TRPV4 can regulate the neurites. For that purpose, DRG neurons were cultured for 36 hours and then treated with a low dose of 4αPDD (0.1 µM) for an additional 36 hours. Neurons were visualized for neuron-specific βIII tubulin. Immunodetection of TRPV4-positive neurons was not conclusive due to the very low endogenous expression of TRPV4 in DRG neurons in this stage. Thus, we categorized the DRG neurons by staining with IB4 lectin, which mark a subsection of nociceptive neurons [49]. We observed that due to long-term exposure of 4αPDD, majority of the IB4-positive neurons developed much shorter neurites if at all (Fig. 4b). In contrast, under the same condition, IB4-negative neurons revealed long neurites similar to non-treated neurons (Fig. 4b). This confirmed that long-term exposure to a TRPV4 agonist resulted in growth cone and neurite retraction, and demonstrates that this effect is restricted to a subpopulation of nociceptors.

### Activation of TRPV4 results in disassembly of microtubules

Previously it has been shown that activation of Ca^2+^ channels causes rapid reorganization of the cytoskeleton [31], [50]–[51]. Also, rapid disassembly of microtubules causes growth cone retraction [32], [34], [52]. So, we tested if in contrast to a stabilizing function of TRVP4 at resting stage, activation of this channel leads to destabilization of microtubules. To better differentiate between soluble and polymerized cytoskeleton components, soluble proteins were removed by Digitonin-extraction leaving behind only the insoluble intact cytoskeleton. Extraction of unstimulated TRPV4 expressing F11 cells showed fine filamentous microtubules (Fig. S5a). But application of 4αPDD (1 µM) resulted in loss of microtubules from the majority of TRPV4 expressing F11 cells. In contrast, cells, which did not express TRPV4 were unaffected by 4αPDD (Fig. S5a).

We observed that the cytoskeleton rearrangement by TRPV4 was not restricted to the neuronal cells only. In TRPV4 expressing CHO-KI cells 4αPDD treatment induced loss of almost all microtubules whereas non-transfected cells retained all their microtubules (Fig. S5b). Similarly, we observed loss of all peripheral microtubules by application of 4αPDD in CHO-KI-TRPV4 cells, which express low level of TRPV4 stably (Fig. 4c). Under the same conditions, CHO-KI mock-transfected cells retained all microtubules. All these results confirm that activation of TRPV4 results in microtubules disassembly and this effect is independent of TRPV4 expression level in whatever cellular context chosen.

### Activation of TRPV4 results in formation of elongated filopodia

**Figure 5:**
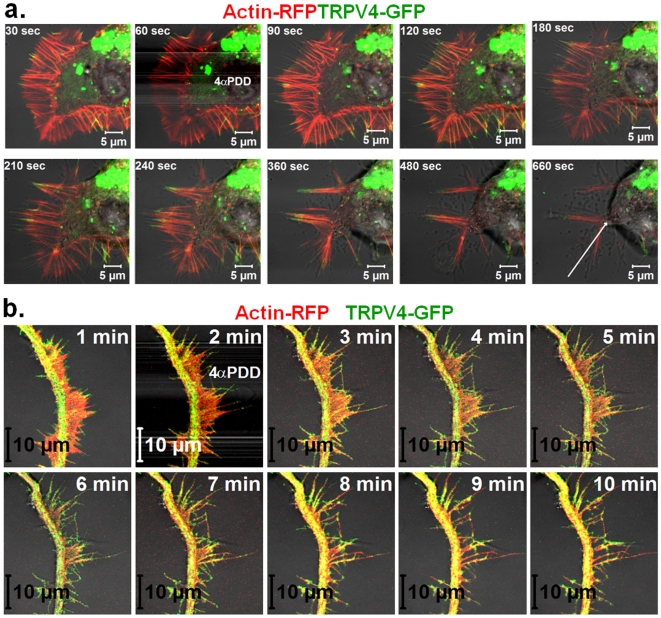
Activation of TRPV4 results in reorganization of actin cytoskeleton. Shown are the confocal images of live F11 cells expressing TRPV4-GFP (green) and Actin-RFP (red). **a.** Activation of TRPV4 results in merging of several actin-ribs at the tips and further transition of lamellipodial structures to filopodial structures. The arrow indicates the region and direction of the cell retraction at the same time. **b.** Activation of TRPV4 results in lateral initiation of filopodial structures from neurites and further elongation of them.

We also explored if TRPV4, being present at the filopodia can alter the actin cytoskeletal dynamics upon activation. For that purpose, we expressed TRPV4-GFP and actin-red in F11 cells and monitored the filopodial structures. We observed that activation of TRPV4 by 4αPDD results in rapid change of lamellipodia to filopodia and/or further elongation of filopodial structures (Fig. 5). These changes were observed at filopodial structures emanating from the cell body as well from neurites. We observed that during this change, several lamellipodial actin ribs fused into single filopodial structures and TRPV4-GFP localized at the filopodial tips (Fig. 5b). At the same time, lamellipodial region located between two actin-ribs retract.

### Stabilization of microtubules by Taxol reduces Ca^2+^-influx through TRPV4 activation

**Figure 6:**
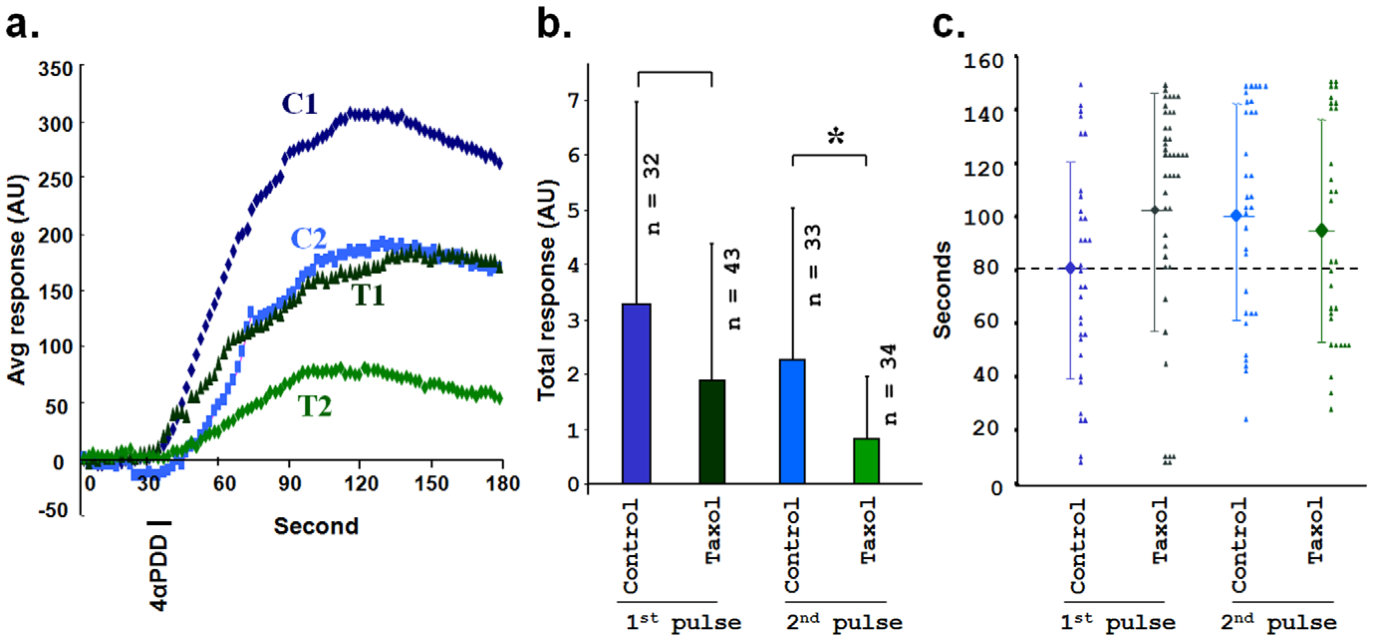
Microtubule cytoskeleton regulates Ca^2+^-influx via TRPV4. **a.** Taxol (1 µM, 30 minutes) reduces TRPV4-mediated Ca^2+^-influx in Cos7 cells. Shown are the normalized average ratiometric Ca^2+^-influx in arbitrary units (AU). TRPV4 was activated by a pulse (indicated by a black line) of 4αPDD for 10 sec. The dark blue line (C1) represents average response due to first pulse in control conditions (n = 32), the light blue line (C2) represents average response due to second pulse in control conditions (n = 32), the blackish green line (T1) represents average response due to first pulse under the influence of Taxol-stabilized microtubules (n = 42) and the light green line (T2) represents the average response due to second pulse under the Taxol-stabilized microtubules (n = 30). **b.** Cells with Taxol-stabilized microtubules reveal reductions in total Ca^2+^-influx due to TRPV4 activation. Total Ca^2+^-influx was calculated from the total area appeared by the ratiometric calcium-influx graph (as shown in figure a) for each cell and was calculated by Origin 7G software. At the <0.05 level, the difference of the population means between 2^nd^ pulse (untreated and taxol-treated, one sample t-test) is significant (*). The difference in case of 1^st^ pulse is non-significant. **c.** Taxol stabilized cells reveal a trend for time delay to reach in maximum response. The time (in seconds) each cell took to reach its maximum response (indicated by filled triangles) was plotted. Times needed during first pulse in control conditions (dark blue), second pulse in control conditions (light blue), first pulse with Taxol (blackish green) and second pulse with Taxol (light green) are indicated. The average values (indicated by filled squares) and the standard deviations are also shown for each condition. The average time differences remain non-significant.

In contrast to a unidirectional effect of TRPV4 activation on microtubule integrity, we explored if microtubule dynamics can also affect the TRPV4 channel properties. We measured Ca^2+^-influx via TRPV4 as an indicator of TRPV4 channel opening. We compared the Ca^2+^-influx via TRPV4 in response to 4αPDD in the presence and absence of Taxol. We observed that the normalized average Ca^2+^-influx due to TRPV4 activation was strongly diminished (but not abolished) if the cells were treated with Taxol (Fig. 6a). This suggests a partial inhibition of TRPV4 channel activity due to stabilization of microtubules. This reduction due to Taxol treatment (30 min) was also observed when cells were activated by 4αPDD for a second time (Fig. 6a). To understand the extent of reduction, we compared the total Ca^2+^-influx (Fig. 6b). We observed 21.9% reduction in the case of the 1^st^ pulse in presence of Taxol when compared to Taxol-free control condition. However, this difference remain non-significant at (P>0.05) (Fig. 6b). However, this difference was even higher in the case of the 2^nd^ pulse where a major reduction (61.79%) was observed in the presence of Taxol (compared with Taxol-free 2^nd^ pulse condition). At the level of P<0.05, this difference is significant. To confirm this inhibitory effect of microtubule stabilization on TRPV4 channel opening, we analysed by another parameter, i.e. the time each cell took to reach its maximum response. We observed that Taxol-treatment resulted in a delay of the cells to reach their maximum response level (Fig. 6c). Though this time difference indicates a clear trend of inhibition, the differences remain non-significant (Fig. 6c). By immunofluorescence analysis (Fig. S6) and surface-biotinylation experiments (Fig. S7), we confirmed that the presence of microtubules and TRPV4 at the cell membrane or nearby regions was also not altered by Taxol application. Taxol did not alter the distribution of TRPV4 in the cell surface also. Taken together, these results suggest that microtubule dynamics can regulate TRPV4 channel properties.

## Discussion

A substantial amount of work has been done to identify endogenous and exogenous sensitizing stimuli, their target proteins and elucidate their molecular effects on nociceptive neurons. Yet, the detailed cellular and molecular mechanisms underlying sensitization against mechanical stimuli and the specific molecular components associated with these signaling events are not well understood. A priori, as there is no force without counterforce, scaffold structures like the cytoskeleton and/or the extracellular matrix are intuitively likely to be required for stimulus detection and execution of the responses. In nociception, so far the cytoskeleton has been described as an important regulator of sensitization only on a behavioural level [29], [42], [53]–[58]. In contrast, in systems other than nociception, genetic, biochemical, cellular as well as behavioural data are indicative of the importance of the cytoskeleton for mechanosensation in general [29], [59]–[60]. Thus, contrasting the wealth of data in non-nociceptive context, the nearly complete lack of molecular data about an involvement of the microtubule and actin cytoskeleton in pain sensitization is surprising. Though, recent results demonstrated the expression of TRPV4 in Merkel cells and linked TRPV4 function with the mechanotransduction [61], [62], in the context of nociceptive and non-nociceptive mechanosensitivity, a direct physical interaction of actin or microtubule cytoskeleton with ion channels has not been reported. Therefore, we now provide the first evidence that both actin and microtubule cytoskeleton components interact directly and functionally with a mechanosensitive ion channel, namely TRPV4.

### The C-terminus of TRPV4 interacts directly with actin and microtubule cytoskeletal components

The C-terminus of various TRP channels has been shown to be of importance. For example, a recent study reported that the deletion of the C-terminal cytoplasmic domain of TRPV4 strongly inhibits membrane localization [63]. And also the C-terminus of TRPC4 interacts with another cytoskeletal component, spectrin, which is essential for the surface expression of the receptor [64]. In this work we now demonstrate that TRPV4-Ct is sufficient for the interaction with tubulin and actin as well as signaling-components. The interaction of MBP-TRPV4-Ct to tubulin and actin appears to be very strong as these interactions withstand the presence of high salt concentrations (500 mM NaCl, data not shown). If the N-terminal cytoplasmic domain of TRPV4 can also interact with the cytoskeleton has not been established yet.

We observed that TRPV4 co-localized with actin-enriched structures, such as focal adhesion points, filopodial and lamellipodial structures. These results fit well with the previously described subcellular localizations of TRPV4 in actin cytoskeleton-enriched structures like dendritic spines [65]. This also accords well with the reported presence of α2 integrin, an actin binding protein in the TRPV4 complex [66]. We show that TRPV4 physically interacts with soluble actin as well as with polymerised actin filaments via its C-terminal cytoplasmic domain. Thus, our biochemical study confirms and extends a recent fluorescence resonance energy transfer (FRET) analysis performed on live cells demonstrating close proximity of actin and TRPV4 [67]. Interestingly, a recent study shows that disruption of the actin cytoskeleton increases the intracellular mobility of TRPV4-GFP and results in loss of co-localization of TRPV4 with actin [68]. Our findings suggest that the direct interaction of TRPV4 and actin is a general feature observed in various cellular systems. This indicates the importance of the interaction far beyond the nociceptive system.

Recent EM study also reveals that TRPV4 forms a “hanging gondola”-like structure which undergoes different structural conformations representing open and closed state of the channel [69]. According to this structure, the C-terminus of TRPV4 is exposed outside and accessible for interaction with other proteins. In that context, it is tempting to speculate that interaction of TRPV4 with the actin and tubulin may have an importance for such conformational changes. It is also important to note that the presence of GFP tag (240 amino acid) at the C-terminus of TRPV4 retains perfect co-localization of TRPV4-GFP with actin and microtubule cytoskeleton, indicating that the tubulin-binding region located with the TRPV4-Ct is most likely not present at the extreme C-terminal free end of the TRPV4. Previously, we identified two novel tubulin-binding motifs, which we mapped to the C-terminal cytoplasmic domain of TRPV1 [36]–[37]. They consist of two short basic amino acid-rich sequence stretches, which are thought to interact directly with the acidic overhangs of tubulin. Indeed, positively charged amino acids in one of these two novel motifs are partially conserved in the C-termini of TRPV4 and TRPV2 [37]. Though we did not perform deletion mapping, based on other experimental evidences and best of our understanding, we applied a similar conceptual basis and predicted that the basic amino acid residues present within the C-terminus of TRPV4, especially within the 746 to 779 amino acids may interact with tubulin (Fig. S1). Though the ability of this small region to interact with tubulin needs to be experimentally verified, we found that indeed the total C-terminus of TRPV4 interacts with tubulin and/or microtubules. Thus, beyond the reported indirect binding through MAP7 [70], we find direct interaction of TRPV4 to microtubule cytoskeleton. In this study we have not tested if the N-terminal region of TRPV4 can interacts with actin and tubulin. However, considering the fact that the N-terminus of TRPV4 contains multiple ankyrin repeats which are presumably important for protein interaction possible interaction of tubulin and actin at the N-terminus cannot be ruled out completely. In this context, it is worth mentioning that recent studies demonstrate that point mutations located at these ankyrin repeats at the N-terminus either increase or decrease the TRPV4 activity and results in genetic disorders [71]–[73].

In this work we investigated if TRPV4 can affect microtubule properties. Binding of different post-translationally modified tubulins suggests that TRPV4 can modulate the stability of the microtubules. Indeed, we show that TRPV4 stabilizes microtubules at the membrane. These microtubules at the membrane can be stained by YL1/2 antibody, which is specific for “tyrosinated tubulin”, indicating that these microtubules are dynamic in nature [74]–[75]. How TRPV4 stabilizes microtubules *in vivo* is not clear. But our *in vitro* work indicates that the C-terminus of TRPV4 is sufficient for microtubule stabilization. The extent of microtubule stabilization by TRPV4-Ct is apparently very strong as TRPV4-Ct stabilizes microtubules are resistant against potent microtubule destabilizers such as Nocodazole and Ca^2+^. Thus, TRPV4-Ct induces “Nocodazole-resistant microtubules”, a class of microtubules that has very different physico-chemical properties [76]–[77]. Notably, a fraction of “Nocodazole-resistant microtubules” are known to be resistant against Ca^2+^ and cold-induced depolymerization [78]–[79]. The resistance of a subset of microtubules against calcium is of high importance at the base of an ion channel, which among others allows calcium influx if activated. The observed binding of post-translationally modified tubulin such as acetylated tubulin, detyrosinated tubulin and polyglutamylated tubulin to TRPV4 may also explain the stabilization as these tubulins are known to be involved in the formation of stable microtubules [76], [80]–[81].

### Regulation of growth cone, neurites and filopodia by TRPV4

Cellular and neuritic morphology is modulated through mutual regulation of the actin and microtubule cytoskeleton. The microtubule cytoskeleton provides an anterograde force while the actin cytoskeleton provides retrograde force. Thus these two opposing forces regulate the morphology and determine the net movement of neurites [32]–[34], [52], [82]. As TRPV4 modulates actin as well as microtubule stability, it has the ability to steer growth cone movements. This result matches well with our previous work demonstrating that activation of TRPV1 causes rapid disassembly of microtubules and thus regulates growth cone movement [31]–[32], [51], [82]. However, unlike TRPV1, TRPV4 activation-mediated (by using 4αPDD) disassembly of microtubules in DRG neurons, in transfected F11 cells as well as in non-neuronal cells seems to be much slower. Therefore, TRPV1 and TRPV4 might be involved in regulation of the neuronal architecture not only in response to different stimuli but also along a different timescale.

### Regulation of TRPV4 activity by actin and microtubule cytoskeleton

TRPV4 not only changes the cellular cytoskeleton but the cellular cytoskeleton is in turn also altering the TRPV4 channel properties. On one hand TRPV4 regulates stability-instability of the microtubules and reorganizes them, on the other hand microtubule dynamics regulate the Ca^2+^-influx through TRPV4. We now show that stabilization of microtubules by Taxol *in vivo* results in reduction and delay in TRPV4-mediated Ca^2+^-influx. Though the values are not statistically significant, these differences show a clear trend of Taxol-mediated inhibition of TRPV4. This result accords well with the fact that Taxol reduces TRPV4-mediated currents in transfected CHO cells [70]. Along the same line, evidence suggests that the actin cytoskeleton can also regulate the TRPV4 channel activity as disruption of the actin cytoskeleton impairs the TRPV4-mediated currents [70] and Ca^2+^-influx in TRPV4 expressing cells in response to hypotonic shock [68].

However, the molecular mechanism underlying this inhibitory effect of Taxol on TRPV4-mediated Ca^2+^-influx is not yet clear. Unlike the response to microtubule destabilizers (like Nocodazole), the integrity of microtubule cytoskeleton, microtubule-based transport and membrane distribution of TRPV4 are not compromised by this short-term Taxol application. We predict that this modulation is most likely due to the conformational change of the channel complex induced by the dynamic presence or absence of the microtubule cytoskeleton and its associated components, which correlates with either channel closure and/or desensitisation. In agreement with this concept, it is important to note that activation of single TRPV4 ion channel in response to heat is possible in whole cell recordings but not in a cell-free inside-out patch clamp experiments, suggesting that in the latter some cellular factor is missing [83]. In this regard it is tempting to speculate an involvement of microtubules there. While in the course of the excision still some microtubules might be attached to the TRPV4 complex, later on it might disassemble easily as filamentous microtubules are sensitive to dilution [84]. However, further single channel electrophysiological investigations are required.

### TRPV4 and cytoskeleton in the context of intracellular pain signaling

Previously it has been shown that potentiation, spontaneous activity and desensitisation of TRPV4 are Ca^2+^-dependent processes, suggesting Ca^2+^-dependent signalling occurs [44], [85]–[86].Indeed, TRPV4 interacts with calmodulin in a Ca^2+^-dependent manner [44]. In agreement with that, we also observed the presence of CamKII in the complex formed by TRPV4-Ct, but only in the presence of Ca^2+^ (Fig. 1c). This is in line with a reported role of CamKII in pain [46]. It also indicates that CamKII may regulate TRPV4 function. However, not all signalling molecules associate with TRPV4 in a Ca^2+^-dependent manner. PKCε, a Ca^2+^-independent kinase investigated in various aspects of mechanical sensitization [39]–[42], [87]–[88] interacts with the C-terminus of TRPV4 in the presence as well as absence of Ca^2+^. Our results are also in line with a recent report demonstrating that TRPV4 phosphorylation at its N-terminus is important for its activation and PKCs along with PKA are important for this phosphorylation [45]. The same report demonstrates that PKCs form a complex with the TRPV4 in the presence of AKAP proteins which function as scaffolds. Considering the fact that AKAP proteins interact with tubulin [89], our results suggest that the complex we identified has a mechanistic function. Though, direct phosphorylation of the C-terminus of TRPV4 by PKCε *in vitro* has not been shown so far, but PKC-mediated potentiation of TRPV4 has been observed [1], [43] and PKCε-dependent activation of TRPV4 has been described *in vivo* [90]. In our preliminary *in vitro* experiments too, we failed to observe any phosphorylation mediated by recombinant (and purified) PKCε when purified MBP-TRPV4-Ct was used as a substrate (data not shown). However, a recent study has demonstrated that *in vivo* condition, S824 position of the TRPV4 is phosphorylated by PKC [91]. However, this phosphorylation might be mediated by other PKC isotypes and may not be by PKCε. Based on our results and data from others, we believe that PKCε most likely sequesters at the C-terminus of the TRPV4 in order to phosphorylate the N-terminus and/or it needs other factors. Nevertheless the complex we isolated in this work appears to be of importance, as cytoskeletal components, CamKII, PKCε and TRPV4; all are known to be involved in pain sensitization [46], [58], [90].

TRPV4 and microtubule cytoskeleton also share similarities in terms of regulation by Sac tyrosine kinase, a pro-nociceptive regulator at the plasma membrane. On a cellular level, Src tyrosine kinase activity in response to mechanical force is localized mainly at the membrane [53]. In accordance with that, Src tyrosine kinase phosphorylates membrane-associated tubulin of neuronal origin [92]. This kinase activity is important for TRPV4 function [66] as it phosphorylates TRPV4 [43], [93]. Thus membrane-associated tubulin-TRPV4 complex seems to be important for stimuli that activate Src-tyrosine kinase.

### Microtubules and TRPV4 in pain sensitization

Involvement of cytoskeleton in pain sensitisation has been anticipated for a long time, as microtubule-based chemotherapeutics against cancer are known to induce severe long-lasting neuropathic pain [94]. But, how these chemotherapeutics, like Taxol and Vincristine alter pain sensitivity is not well understood. Microtubules and mitochondrial structures develop abnormalities in nociceptive neurons due to prolonged and systemic application of these chemotherapeutics [95]–[97]. Even stabilization of microtubules by short-term application of Taxol (30 min) exerts an impact on pain sensitization [58]. It is important to mention that disruption of microtubules by short-term (30 min) application of Nocodazole abolishes/reduces pain sensitization [58]. These suggest that the integrity of microtubules can indeed modulate pain sensitivity [98]. TRPV4 has recently been considered as essential for chemotherapy-induced neuropathic pain as mechanical hyperalgesia induced by Taxol and Vincristine was strongly reduced in *trpv4* −/− mice [30], [66].

We established a direct physical, cell-biological and functional relationship between cytoskeletal components and TRPV4, which plays an important role in inflammatory, neuropathic and chemotherapeutic-induced pain [30], [58], [66]. This gives a mechanistic base to further study the role of the apparent dynamic signalling complex formed by TRPV4 and its functional implications in various physiological scenarios.

## Materials and Methods

### Reagents and antibodies

Taxol, Nocodazole, 4αPDD, bovine actin, tetramethylrhodamine isothiocyanate-labelled IB4 from *Griffonia simplicifolia*, antibodies against α-tubulin (clone DM1A), β-tubulin (clone D66), tyrosinated tubulin (clone TUB1A2), polyglutamylated tubulin (clone B3), acetylated tubulin (clone 611-B-1), phospho-serine (Clone PSR-45), β-tubulin sub type III (clone SDL.3D10), neurofilament 116 kDa (clone NN18) and the affinity purified rabbit polyclonal antibody against C-terminal cytoplasmic domain of TRPV4 were purchased from Sigma-Aldrich (Taufkirchen, Germany). Antibodies against neurofilament 200 kDa (clone RT97) and detyrosinated tubulin were purchased from Chemicon (Chandlers Ford, UK). The antibody against actin (clone JLA20) was purchased from Oncogene (Cambridge, MA, USA). Antibody against maltose-binding protein (MBP) and the amylose resin were purchased from New England Biolab (Beverly, MD, USA). Purified bovine muscle actin was purchased from Sigma-Aldrich (Taufkirchen, Germany). Protein G-agarose was purchased from Amersham Pharmacia Biotech (Munich, Germany). For some experiments, another affinity purified rabbit polyclonal antibody raised against TRPV4 (kind gift from Jon D. Levine) was used [3]. Anti CamKII antibody was purchased from BD (Heidelberg, Germany). Anti PKCε antibody (KP4) was a kind gift from Dr Robert Messing, University of California San Francisco. All secondary IgG antibodies (alexa-488-labelled anti-mouse, alexa-488-labelled anti-rabbit, alexa-594-labelled anti-rabbit, alexa-594-labelled anti-rat, Fura-2/AM, alexa-488- and alexa-594-labelled Phalloitoxin were purchased from Invitrogen (Karlsruhe, Germany).

### Constructs

Full-length TRPV4 (NCBI accession number AF263521) in pCDNA3.1 vector was a kind gift from Prof. Jon D Levine [3]. For expression of C-terminal cytoplasmic domain (amino acid residue 718 to 871) of TRPV4-fused with MBP, the corresponding cDNA region of TRPV4 (NCBI accession number AF263521) was amplified by using forward primer (5′TGACGAATTCATGGGTGAGACCGTGGGCCA3′) and reverse primer (5′TGACAAGCTTCTACAGTGGTGCGTCCTCCG3′). The amplified cDNA was sub cloned into the EcoRI and HindIII restriction site of the pMALc2x vector (NEB, Beverly, MD, USA) and verified by automated nucleotide sequencing. TRPV4-GFP construct (cloned in pEGFPN3 vector) used in this study was kindly provided by Prof. J. Berreiter-Hahn [10]. Tubulin-cherry construct was a kind gift from Prof. R. Y. Tsien [99]. RFP-actin construct was purchased from Clontech (Heidelberg, Germany).

### Cells and transfection

F11 cells [38] were cultured in Ham's F12 medium (Sigma Aldrich) supplemented with 20% fetal bovine serum (Sigma Aldrich). Cos7 [100] and HaCat [101] cells were maintained in Dulbecco's modified Eagle's medium (Sigma Aldrich) with 10% fetal calf serum. CHO-K1 cells [3] stably transfected with TRPV4 (CHO-K1-TRPV4) and the negative control cell line (CHO-K1-MOCK) [3] was cultured in F12 Ham's (Sigma Aldrich) medium supplemented with 5% fetal bovine serum (FBS) and L-glutamine. These two cell lines were kindly provided by Prof. J. D. Levine [3]. All media were supplemented with streptomycin (100 µg/ml) and penicillin (100 µg/ml) (Invitrogen). Cells were maintained in a humidified atmosphere at 5% CO_2_ and 37°C. For transient transfection, Lipofectamine (Invitrogen) was used according to the manufacturer's instructions.

### Ethics Statement

Cell biological experiments were performed in cultured DRG neurons from male Sprague-Dawley rats (200–300 g; Harlan Winkelmann, Borchen, Germany). Care and use of animals were in accordance with the European Communities Council Directive of 24 November 1986 (86/609/EEC) and were approved by the LaGeSo, Berlin. All efforts were made to minimize the number of animals used and their suffering.

### DRG neuronal culture and neurite out growth

Dissociated rat DRG neurones were prepared from adult male rats (200–300 g) as recently described [32]. Neurons were grown in 24-well plates at 37°C in 5% CO_2_ and were grown for a total of 72 h. At that time most untreated neurons grow numerous and long neurites. In some experiments neurons were treated with 4αPDD (0.1 µM for the last 36 hour) before they were fixed with 2% PFA. Subsequently the neurons were visualized for βIII-tubulin (antibody dilution 1∶1000) and IB4-lectin (1∶15000).

### Immunoprecipitation

For immunoprecipitation, approximately 50 µl of 50% protein G beads-slurry equilibrated with IP buffer (1% sodium dodecyl maltoside, PIPES 50 mM (pH 6.8), 100 mM NaCl, 1 mM EGTA, 0.2 mM MgCl_2_ and complete protease inhibitors (Roche) was used for each IP. Affinity purified rabbit polyclonal TRPV4 antibody (Sigma Aldrich) (7 µg per IP condition, antibody obtained from Sigma-Aldrich) was used. In control, total serum IgG from rabbit (7 µl which is equivalent to 20 µg of antibody) was used as non-specific antibody. For co-immunoprecipitation, CHO-KI-TRPV4 cells grown for 2 days and then scraped off from the dishes. Cells were harvested by centrifuging (Hettich Rotanta/T, 5 min at 100 *g*). Cells were washed with PBS once and centrifuged again at the same conditions. Cells were resuspended in 2 ml of IP buffer and homogenized (10 strokes) with a glass-Teflon homogenizer (Kontes glass, Germany). Cell extract was further centrifuged at 16 K (25000 g) for 30 minute at 4°C and the clear supernatant was used for IP. Similarly, DRG tissues (L1-L6) from 2 male rats were isolated, homogenized in IP buffer. The lysate was centrifuged at 25.000 g, 30 min at 4°C and the clear extract was used for the further IP. Clear cell extract (800 µl equivalent to 1 mg of protein) and the corresponding antibody were applied to the equilibrated protein G beads. The mixture was incubated at 25°C for 4 hours on a shaker. After that the beads were washed 3 times, each time with 400 µl of IP buffer. A Hamilton syringe was used for all washing steps. Finally the beads were taken in 100 µl of IP buffer and 50 µl of 5x Laemmli sample buffer was added. The samples were boiled and used for western blot analysis.

### Expression and purification of MBP-fusion proteins

Expression and purification of MBP-TRPV4-Ct (C-terminal cytoplasmic domain of TRPV4 fused with MBP) as well as of MBP-LacZ (LacZ fused with MBP) was based on a protocol described previously [36]. In short, the expression constructs were introduced into the *Escherichia coli* (*E. coli*) strain BL21DE3 by transformation heat shock method. Fusion protein expression was induced by addition of isopropyl thiogalactoside (IPTG) for 2 h. Cells were lysed by repeated freeze-thaw cycles in lysis buffer (20 mM Tris–HCl, pH 7.4, 150 mM NaCl, 0.1% Tween 20, lysozyme, benzonase and protease inhibitor cocktail). The lysed extracts were cleared by centrifugation (100000 *g* in a TFT 45 rotor for 2 h) and applied to amylose resin. The resins with bound proteins were washed thoroughly and finally the proteins were eluted with 10 mm maltose in elution buffer (50 mM PIPES, pH 6.8, 100 mM NaCl, 1 mM EGTA and 0.2 mM MgCl_2_).

### Pull down assays

MBP-LacZ and MBP-TRPV4-Ct constructs were expressed in *E. coli*; the cleared cell lysates were applied to amylose resin (NEB), and incubated for 1 hour at RT (25°C) followed by washing. The amylose resin with bound proteins was resuspended in PEM-S buffer (50 mM PIPES, pH 6.8, 100 mM NaCl, 1 mM EGTA and 0.2 mM MgCl_2_). Approximately 50 µl of amylose resin per tube with the bound fusion protein was used for pull-down experiments. Depending on the respective experiment, the resin with coupled fusion protein was incubated with either 50 µl of soluble tubulin (1 mg/ml protein), 20 µl of soluble actin (0.1 mg/ml), 500 µl of soluble brain extract (1 mg/ml), or 500 µl of soluble F11 extract (1 mg/ml) for 1 hour at RT in the presence or absence of Ca^2+^ (2 mM). This was followed by three washes with 200 ml PEM-S buffer. The proteins were eluted by 10 mm maltose in 100 µl solution. Eluted samples were analysed by 10% sodium dodecyl sulphate polyacrylamide gel electrophoresis (SDS–PAGE).

### Co-sedimentation assay with polymerised actin, Taxol-stabilized microtubules (MT) and analysis for microtubule stability

For experiments aimed at understanding the association of TRPV4-Ct with polymerised actin filaments, purified MBP-TRPV4-Ct alone was subjected to centrifugation at 70000 *g*/30 min/4°C to exclude any aggregates. The soluble MBP-TRPV4-Ct was further processed for all co-sedimentation assays. Either 5 µg of purified MBP-TRPV4-Ct or MBP alone in total 50 µl of PEM-S buffer or only 50 µl of PEM-S buffer was added to a saturated actin solution (50 µg actin in total 50 µl) in centrifuge tubes. ATP at a final concentration of 2.5 mM was added to each tube. The reaction tubes were incubated at 37°C for 30 minutes followed by centrifugal separation of pellet (polymerised actin filaments) and supernatant (free dimer) at 70000 *g*/30 min/37°C.

Approximately 100 µg of purified αβ-tubulin dimer in a total volume of 100 µL were incubated in modified PEM buffer (20 mm PIPES, pH 6.8, 0.2 mm MgCl_2_ and 1 mm EGTA supplemented by 1 µm Taxol and 5 mm GTP) for 30 min at 37°C, to form MTs. After that the MT were isolated by centrifugation at 70 000 *g*/30 min/37°C. Purified MBP-TRPV4-Ct or MBP only (each 5 µg) were incubated with Taxol-stabilized MT for 40 min at 37°C followed by centrifugal separation of pellet (MT) and supernatant (free dimer) at 70 000 *g*/30 min/37°C.

In another experiment, MT was formed under Taxol-free conditions. Tubulin dimer (100 µg) with purified MBP-TRPV4-Ct or MBP only (5 µg each) were taken in total 100 µl PEM-S buffer with 5 mm GTP in the absence of Taxol and incubated for 30 min at 37°C. After that the MT and bound proteins were separated by centrifugal separation of pellet (MT) and supernatant (free dimer) at 70000 *g*/30 min/37°C.

For experiments aimed at understanding the effect of TRPV4-Ct on MT stabilization, either 5 µg of purified MBP-TRPV4-Ct or MBP alone in total 50 µl of PEM-S buffer or only 50 µl of PEM-S buffer was added to a tubulin solution (5 µg tubulin in total 50 µl) in centrifuge tubes. Depending on the experimental conditions, MT-depolymerizing agent Nocodazole (final concentration 10 µM), Ca^2+^ (final concentration 0.1 mM) or both Nocodazole and Ca^2+^ was added to the tubes. GTP at a final concentration of 2.5 mM was added to each tube. The reaction tubes were incubated at 37°C for 30 minute s followed by centrifugal separation of pellet (MT) and supernatant (free dimer) at 70000 *g*/30 min/37°C.

### Purification of tubulin

αβ-tubulin dimers were purified from porcine brain as described previously [36]. In brief, from soluble brain extract tubulin was enriched by two cycles of polymerization in the presence of glycerol and GTP and depolymerization by cold temperature (ice cold), which were then followed by chromatography on phosphocellulose.

### Immunocytochemistry

For immunocytochemical analysis, cells were washed once with PBS and fixed with 2% PFA/PBS if not mentioned otherwise. Alternatively, where mentioned, an equal volume of 4% PFA was carefully added to the medium without disturbing the cell culture within the incubator to avoid morphological changes due to the PBS wash. The cells were fixed with PFA for 5 minutes at room temperature (25°C). For experiments aimed to explore the integrity of the cytoskeleton after TRPV4 activation, soluble components were removed by digitonin extraction as described before [31]. Briefly, after activation the cells were extracted with an isotonic cell membrane-permeabilization buffer (digitonin (50 µg/ml), PIPES (50 mM pH 6.8), EGTA (1 mM), MgCl_2_ (0.2 mM) and complete protease inhibitors (Roche) that perforates the cell membrane and allow all the soluble cellular proteins to diffuse out while insoluble cytoskeleton and cell morphology remain intact. After extraction the cells were quickly fixed with 2% PFA. Fixed cells were permeabilized with 0.1% Triton X-100 in PBS (5 min) and subsequently blocked with 5% normal goat serum or 5% BSA.

For co-immunostaining of TRPV4 and tubulin, a rabbit polyclonal anti TRPV4 antibody (Sigma Aldrich) and a rat monoclonal anti-tyrosinated tubulin antibody (clone YL1/2) were used. For staining of actin filaments, either alexa-594 or alexa-488 labelled Phalloidin (molecular probe) was used. Alexa 594-labelled anti rat antibody and/or alexa 488 labelled anti rabbit antibody were used as secondary antibodies. For labeling IB4-positive neurons, tetramethylrhodamine isothiocyanate-labelled IB4 from *Griffonia simplicifolia* was used at 1∶15000 dilutions in PBST supplemented with 0.2 mM CaCl_2_, 0.2 mM MgCl_2_ and 0.2 mM MnCl_2_ and incubated for 30 minutes. All images were taken on a confocal laser-scanning microscope (Zeiss Axiovert 100 M) with a 63x-objective and analyzed with the Zeiss LSM image examiner software.

### TRPV4 activation and live cell imaging

For visualizing the effect of TRPV4 activation, F11 cells were seeded on glass cover slips. Two days after transfection with TRPV4, the cells in either complete medium or in HBSS buffer were imaged at RT. Where indicated, TRPV4-GFP was co-expressed with either RFP-Actin or Tubulin-cherry. In experiments aiming to activated TRPV4, its agonist 4 α-phorbol 12,13-didecanoate (4αPDD) was used at a final concentration of 1 µm, if not stated otherwise [22].

### Calcium imaging

For measuring the TRPV4 channel activity; Ca^2+^-imaging was performed using Cos7 cells at room temperature (25°C). Cos7 cells plated on glass cover slips and transiently transfected with TRPV4 and a yellow fluorescent protein (YFP) reporter plasmid. Cells transfected with only YFP reporter plasmid were used as controls. The transfected cells were used for the experiments at around 48 hour later transfection. Cells were loaded with 3 µM Fura-2 AM and placed in a recording chamber that allowed direct application of buffer at controlled temperature (27 to 30°C). Loading of Fura-2 AM dye and the fluorescence measurements were done in calcium imaging buffer (140 mM NaCl, 4 mM KCl, 2 mM CaCl_2_, 1 mM MgCl_2_, 5 mM Glucose, 10 mM HEPES, pH 7.4). Cells were activated by 4αPDD (1 µM) for 10 seconds after first 30 seconds of imaging. Images were captured in every 2 seconds for a total of 3 minutes. 10 minutes after the 1^st^ round of activation (1^st^ pulse of 4αPDD) and imaging, cells on the same cover slips were imaged again for the next round (2^nd^ pulse of 4αPDD). In experiments aimed to analyze the effect of microtubule stabilization, Taxol (1 µM) was maintained throughout the experiment in all solutions used, i.e. during the dye loading, during activation by 4αPDD and in between when running calcium-imaging buffer was applied. Ratiometric calcium imaging was performed and analyzed using Tillvision software (Till Photonics, Germany). Fluorescence was measured at excitation wavelengths alternating between 340 nm and 380 nm. Following subtraction of background fluorescence the ratio of fluorescence at 340 nm and 380 nm was calculated. For determination of total Ca^2+^-influx, we measured the total area of the graph each cell formed after stimulation. Microsoft Excel and Origin software were used to calculate and plot the graphs. All significance tests were performed at the <0.05 level (one sample t-test).

## Supporting Information

Figure S1 Prediction of tubulin-binding region located within the C-terminus of TRPV4. a. Identification of amino acid stretches located within the TRPV4-Ct with high isoelectric point (pI) values. The C-terminal overhanging region of tubulin contains highly acidic residues and most of the tubulin-binding proteins contain amino acid stretches which have high pI due to the presence of basic residues. Therefore, the C-terminus of TRPV4 (amino acid residues 718 to 871) was analyzed for its isoelectric points. Similarly, different fragments of TRPV4-Ct, each shorter by 10 amino acids from the extreme C-terminal end were also analyzed. This analysis indicates that there are two areas which contain positively charged residues (indicated by shaded area). The corresponding pI of each fragment is mentioned in the right most side. In below, the secondary structure prediction is provided (red indicates helical structure, green arrow indicates beta strand and black line indicates unstructured coil region. Note that the arrows do not indicate the directionality of the strands). All theoretical pI values of the fragments were calculated by using available software (http://www.expasy.org/tools/pi_tool.html). All secondary structure prediction was done by using Advanced Protein Secondary Structure Prediction Server available at http://imtech.res.in/raghava/apssp/. b. Amino acid residues 746 to 779 contain multiple positively charged amino acids which can potentially form an alpha-helix. The PyMol programme was used to build this peptide structure. Blue and red colours indicate the positive and negative charges at the surface respectively. Notably all the positively charged amino acids (indicated by arrows) are located in one side of the helix and can be important for interaction with the tubulin/microtubule (which contain negative charges at the surface). c. These predicted and important basic charges (indicated by blue) are conserved throughout the evolution. The NCBI accession numbers are indicated. TRPV4, *Homo sapiens* (NP_067638.3); TRPV4, *Canis lupus familiaris* (XP_543434.2); TRPV4, *Bos taurus* (XP_001253150.1); TRPV4, *Mus musculus* (NP_071300.1); TRPV4, *Rattus norvegicus* (NP_076460.1); TRPV4, *Gallus gallus* (NP_990023.1); TRPV4, *Danio rerio* (NP_001036195.1); Putative uncharacterized protein, *Ailuropoda melanoleuca* (PANDA_020862); Putative uncharacterized protein, *Ailuropoda melanoleuca* (PANDA_018294), Chromosome 12 SCAF14999, *Tetraodon nigroviridis* (GSTENG00029525001).
(7.95 MB TIF)

Figure S2 TRPV4 pulls down cytoskeletal components and nociceptive regulator kinases from F11 cell extract. MBP-TRPV4-Ct (lane 2–3) but not MBP-LacZ (lane 4–5) forms specific complex when incubated with soluble F11 extract (lane 1), both in presence (lane 2 and 4) or absence (lane 3 and 5) of Ca^2+^ (1 mM). Presence of actin, tubulin and PKCε are observed only in lane 2 and 3 whereas CamKII is present only in lane 2.
(8.31 MB TIF)

Figure S3 TRPV4 co-localizes with actin cytoskeleton. Shown are the confocal images of neuronal and non-neuronal cells expressing TRPV4. Cells were stained for TRPV4 (green) and actin (red). Presences of TRPV4 specifically in actin-enriched structures (indicated by arrows) are shown. Enlarged views of filopodial structures developed from HaCat cell (upper panel) and F11 (lower panel) cell are shown.
(0.92 MB TIF)

Figure S4 TRPV4 activation can influence the neuritic morphology. Shown are the time-series confocal images of a neurite developed from a F11 cell expressing TRPV4-GFP (green) and Tubulin-cherry (red). This neurite produces varicosities (indicated by arrows) after adding 4αPDD due to disassembly of MT. Note that all the varicosities are formed simultaneously (at 5th minute in this case), indicating a global disassembly of microtubules all over this neurite. Tubulin Cherry construct was a kind gift from Prof. R. Y. Tsien (Shaner et al. 2004).
(0.85 MB TIF)

Figure S5 Activation of TRPV4 results in microtubule disassembly. a. F11 cells were mock activated with buffer or activated with 4αPDD (1 µM) followed by detergent extraction with isotonic buffer and fixed. Cells were immunostained for TRPV4 (green) and tubulin (red). In absence of activation, TRPV4 expressing cells retains all the microtubules (upper panel, i-iii), but lost all the distal microtubules if activated with 4αPDD (middle panel, iv-vi). The stable MTOC region is marked with an arrow. Note that non-transfected cells retain all the microtubules even after addition of 4αPDD (lower panel). b. CHO-KI cells expressing TRPV4 (green) were activated with 4αPDD (1 µM) and were further extracted with detergent in isotonic buffer and fixed. Under this condition, nontransfected cells show microtubules all over the cells, while transfected cells contain very little microtubules.
(1.67 MB TIF)

Figure S6 TRPV4 localization at membrane is not affected by Taxol. Shown are the confocal images depicting the distribution and co-localization of TRPV4 (green) and tubulin (red) in Cos7 cells. Cells expressing TRPV4 were fixed under control condition (i), fixed after incubation with Taxol for 30 minute (ii) or incubation with Nocodazole for 30 minutes (iii). Cells were immunostained for TRPV4 (green) and tubulin (red). The white line indicates the periphery of the cell. All these images were acquired at an identical laser power and gain value.
(1.62 MB TIF)

Figure S7 Microtubule stabilization by Taxol does not alter the surface distribution of TRPV4. To confirm that Taxol-treated cells still contain TRPV4 at the cell surface, we performed surface biotinylation of TRPV4-GFP expressing Cos7 cells that were either treated with Taxol (1 µM, 30 minutes) or left untreated. We isolated TRPV4-GFP by immunoprecipitation and found that TRPV4-GFP is similarly labelled with biotin in both conditions. These results confirm that short-term Taxol application does not alter the distribution of TRPV4 in the membrane. Total cell extract in control condition (lane 1) or under Taxol-stabilized condition (lane 2), TRPV4 immunoprecipitates from control condition (lane 3) or from Taxol-stabilized condition (lane 4) were blotted with TRPV4 antibody (upper panel) and with HRP-Avidin (lower panel). Arrow indicates the position of biotinylated TRPV4.
(1.19 MB TIF)
