# Supplementary figures and images for: Importance of Non-Selective Cation Channel TRPV4 Interaction with Cytoskeleton and Their Reciprocal Regulations in Cultured Cells
Source: PLoS One. 2010 Jul 19;5(7):e11654. doi: 10.1371/journal.pone.0011654 (PMC2906515; doi:10.1371/journal.pone.0011654)

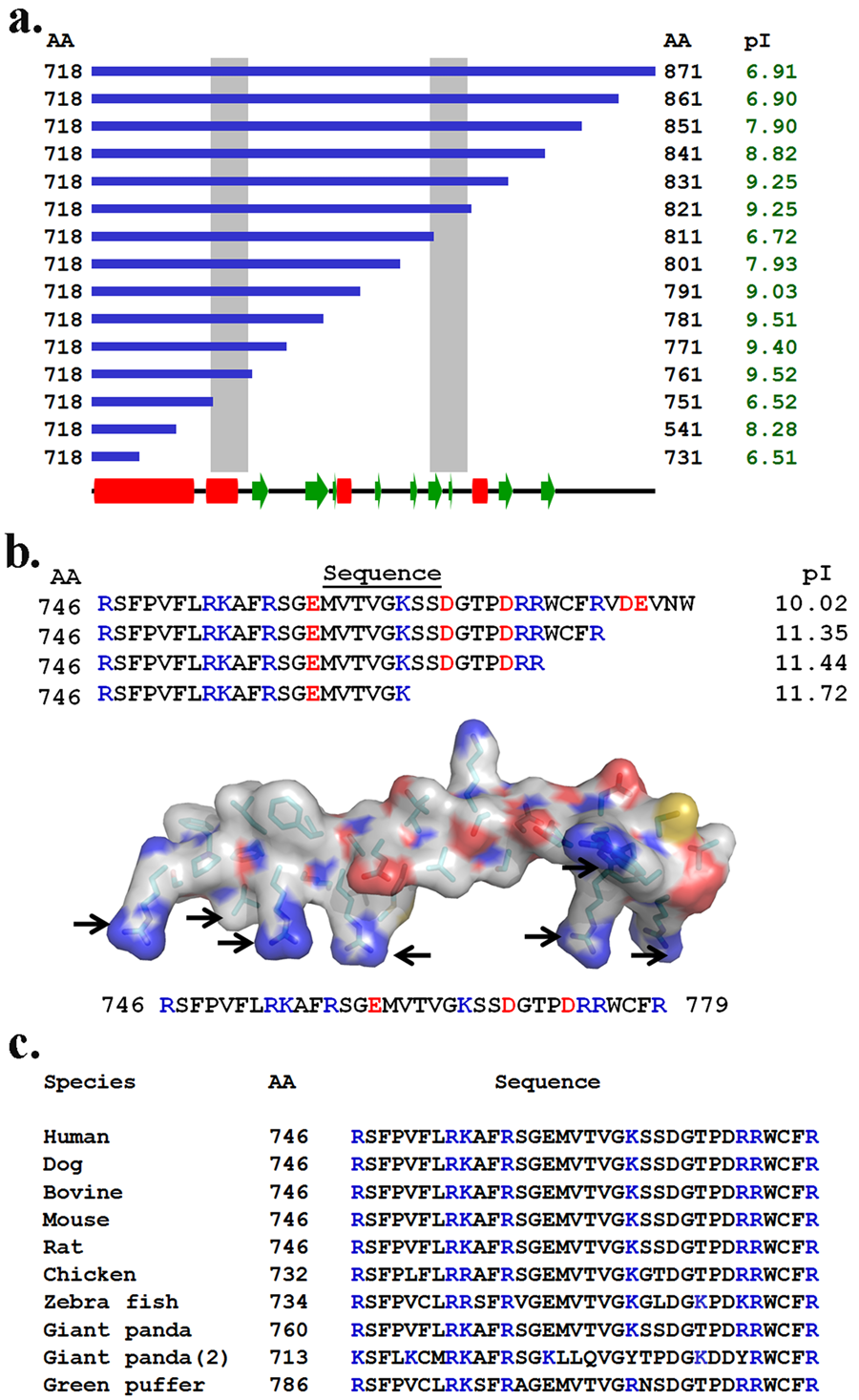

Supplement: Figure S1 — Prediction of tubulin-binding region located within the C-terminus of TRPV4. a. Identification of amino acid stretches located within the TRPV4-Ct with high isoelectric point (pI) values. The C-terminal overhanging region of tubulin contains highly acidic residues and most of the tubulin-binding proteins contain amino acid stretches which have high pI due to the presence of basic residues. Therefore, the C-terminus of TRPV4 (amino acid residues 718 to 871) was analyzed for its isoelectric points. Similarly, different fragments of TRPV4-Ct, each shorter by 10 amino acids from the extreme C-terminal end were also analyzed. This analysis indicates that there are two areas which contain positively charged residues (indicated by shaded area). The corresponding pI of each fragment is mentioned in the right most side. In below, the secondary structure prediction is provided (red indicates helical structure, green arrow indicates beta strand and black line indicates unstructured coil region. Note that the arrows do not indicate the directionality of the strands). All theoretical pI values of the fragments were calculated by using available software (http://www.expasy.org/tools/pi_tool.html). All secondary structure prediction was done by using Advanced Protein Secondary Structure Prediction Server available at http://imtech.res.in/raghava/apssp/. b. Amino acid residues 746 to 779 contain multiple positively charged amino acids which can potentially form an alpha-helix. The PyMol programme was used to build this peptide structure. Blue and red colours indicate the positive and negative charges at the surface respectively. Notably all the positively charged amino acids (indicated by arrows) are located in one side of the helix and can be important for interaction with the tubulin/microtubule (which contain negative charges at the surface). c. These predicted and important basic charges (indicated by blue) are conserved throughout the evolution. The NCBI accession numbers are [file pone.0011654.s001.tif]

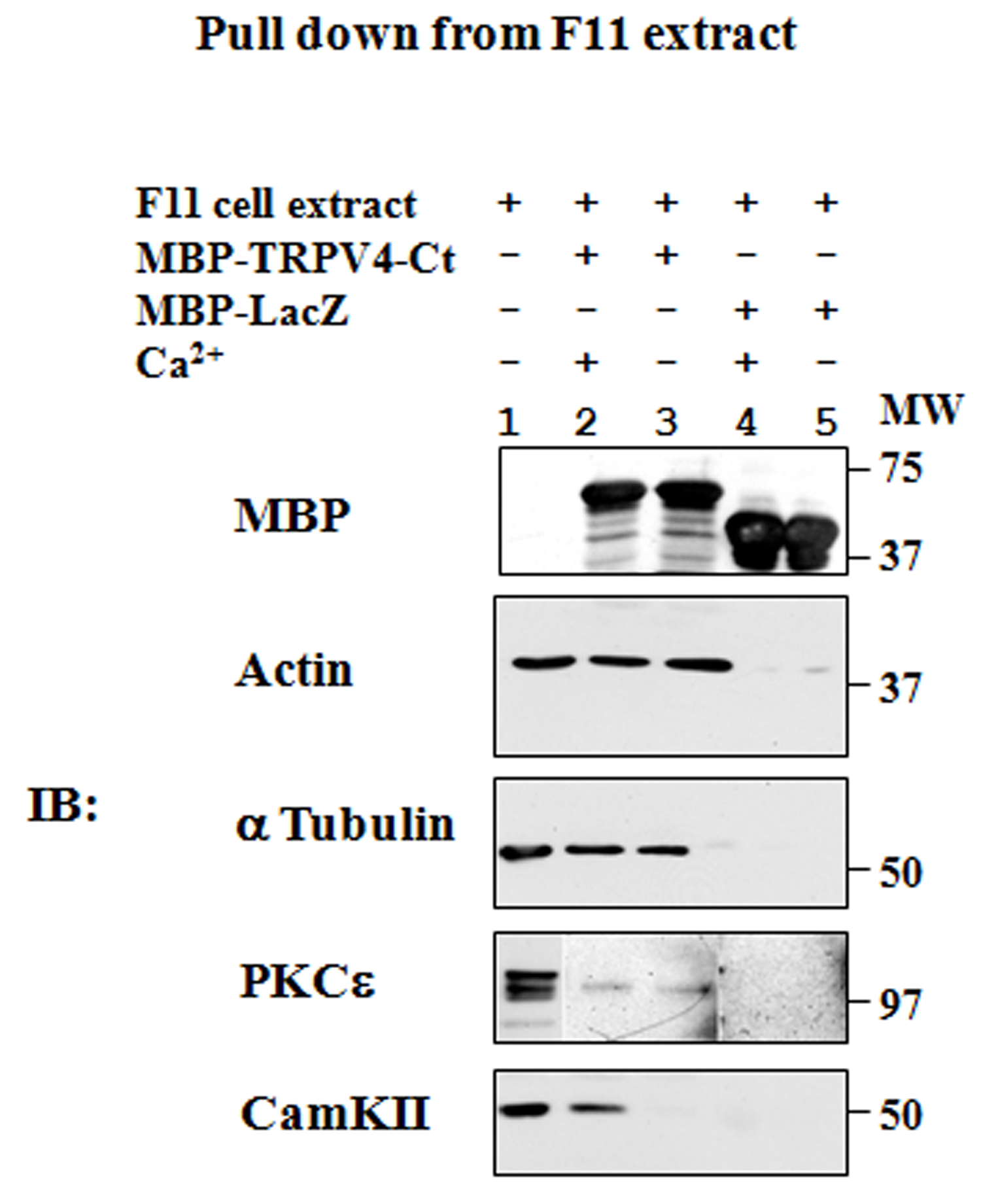

Supplement: Figure S2 — TRPV4 pulls down cytoskeletal components and nociceptive regulator kinases from F11 cell extract. MBP-TRPV4-Ct (lane 2–3) but not MBP-LacZ (lane 4–5) forms specific complex when incubated with soluble F11 extract (lane 1), both in presence (lane 2 and 4) or absence (lane 3 and 5) of Ca2+ (1 mM). Presence of actin, tubulin and PKCε are observed only in lane 2 and 3 whereas CamKII is present only in lane 2. (8.31 MB TIF) [file pone.0011654.s002.tif]

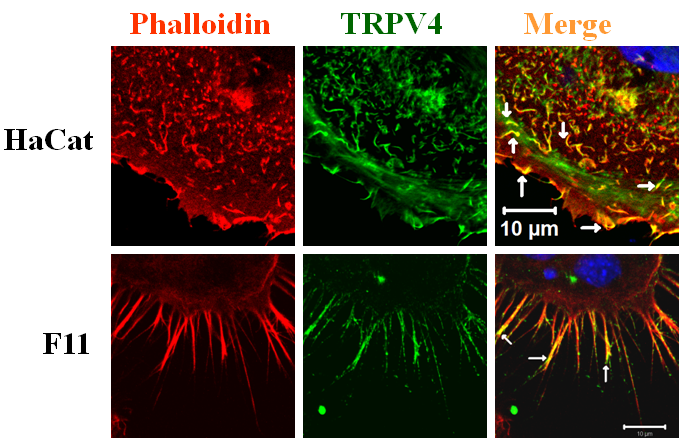

Supplement: Figure S3 — TRPV4 co-localizes with actin cytoskeleton. Shown are the confocal images of neuronal and non-neuronal cells expressing TRPV4. Cells were stained for TRPV4 (green) and actin (red). Presences of TRPV4 specifically in actin-enriched structures (indicated by arrows) are shown. Enlarged views of filopodial structures developed from HaCat cell (upper panel) and F11 (lower panel) cell are shown. (0.92 MB TIF) [file pone.0011654.s003.tif]

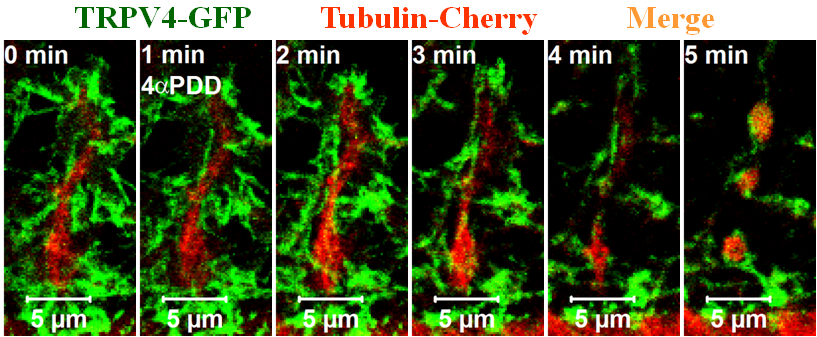

Supplement: Figure S4 — TRPV4 activation can influence the neuritic morphology. Shown are the time-series confocal images of a neurite developed from a F11 cell expressing TRPV4-GFP (green) and Tubulin-cherry (red). This neurite produces varicosities (indicated by arrows) after adding 4αPDD due to disassembly of MT. Note that all the varicosities are formed simultaneously (at 5th minute in this case), indicating a global disassembly of microtubules all over this neurite. Tubulin Cherry construct was a kind gift from Prof. R. Y. Tsien (Shaner et al. 2004). (0.85 MB TIF) [file pone.0011654.s004.tif]

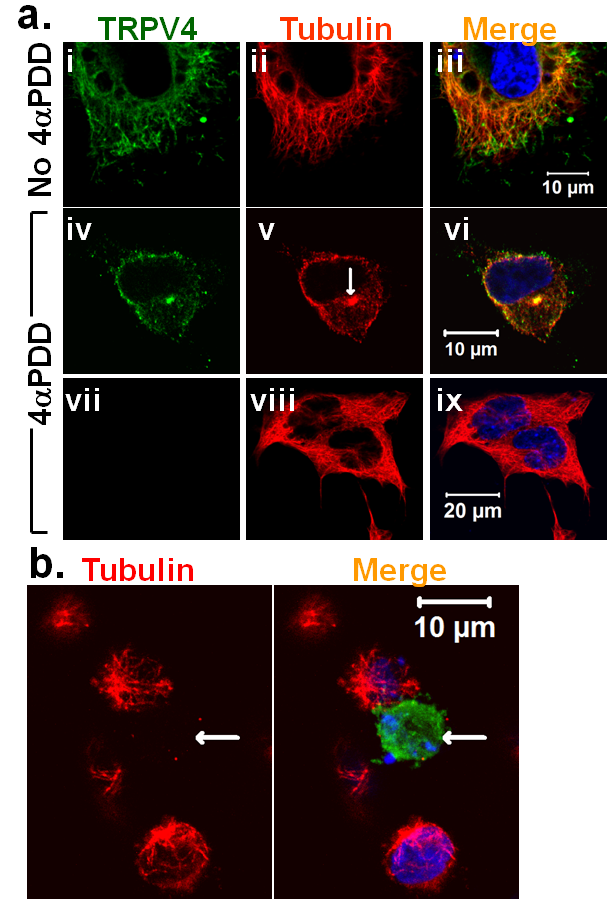

Supplement: Figure S5 — Activation of TRPV4 results in microtubule disassembly. a. F11 cells were mock activated with buffer or activated with 4αPDD (1 µM) followed by detergent extraction with isotonic buffer and fixed. Cells were immunostained for TRPV4 (green) and tubulin (red). In absence of activation, TRPV4 expressing cells retains all the microtubules (upper panel, i-iii), but lost all the distal microtubules if activated with 4αPDD (middle panel, iv-vi). The stable MTOC region is marked with an arrow. Note that non-transfected cells retain all the microtubules even after addition of 4αPDD (lower panel). b. CHO-KI cells expressing TRPV4 (green) were activated with 4αPDD (1 µM) and were further extracted with detergent in isotonic buffer and fixed. Under this condition, nontransfected cells show microtubules all over the cells, while transfected cells contain very little microtubules. (1.67 MB TIF) [file pone.0011654.s005.tif]

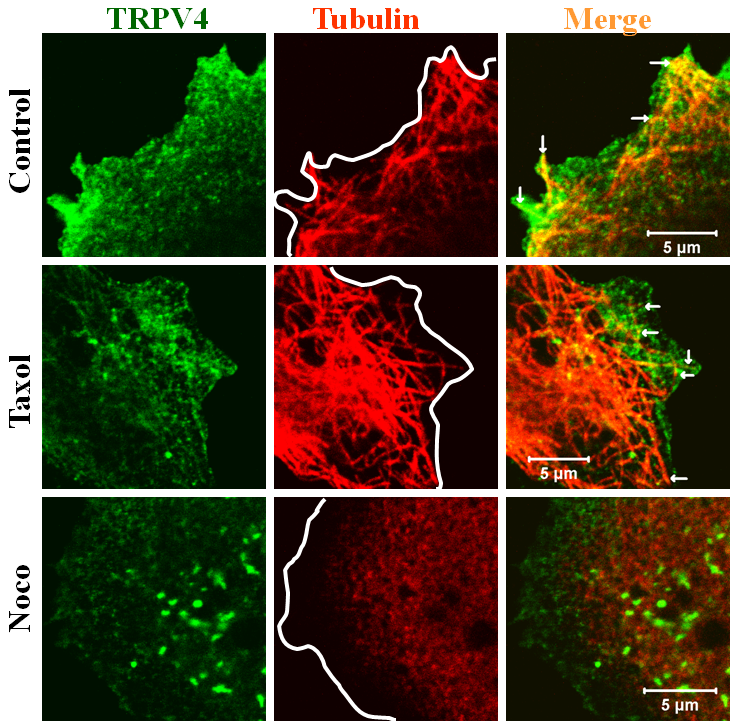

Supplement: Figure S6 — TRPV4 localization at membrane is not affected by Taxol. Shown are the confocal images depicting the distribution and co-localization of TRPV4 (green) and tubulin (red) in Cos7 cells. Cells expressing TRPV4 were fixed under control condition (i), fixed after incubation with Taxol for 30 minute (ii) or incubation with Nocodazole for 30 minutes (iii). Cells were immunostained for TRPV4 (green) and tubulin (red). The white line indicates the periphery of the cell. All these images were acquired at an identical laser power and gain value. (1.62 MB TIF) [file pone.0011654.s006.tif]

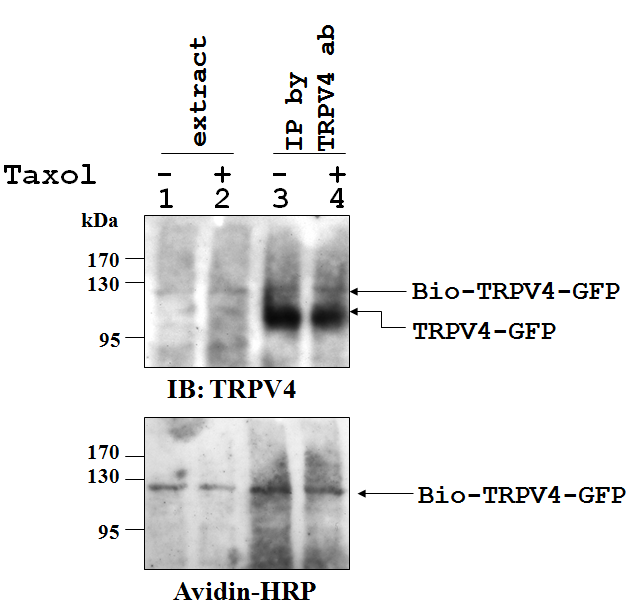

Supplement: Figure S7 — Microtubule stabilization by Taxol does not alter the surface distribution of TRPV4. To confirm that Taxol-treated cells still contain TRPV4 at the cell surface, we performed surface biotinylation of TRPV4-GFP expressing Cos7 cells that were either treated with Taxol (1 µM, 30 minutes) or left untreated. We isolated TRPV4-GFP by immunoprecipitation and found that TRPV4-GFP is similarly labelled with biotin in both conditions. These results confirm that short-term Taxol application does not alter the distribution of TRPV4 in the membrane. Total cell extract in control condition (lane 1) or under Taxol-stabilized condition (lane 2), TRPV4 immunoprecipitates from control condition (lane 3) or from Taxol-stabilized condition (lane 4) were blotted with TRPV4 antibody (upper panel) and with HRP-Avidin (lower panel). Arrow indicates the position of biotinylated TRPV4. (1.19 MB TIF) [file pone.0011654.s007.tif]
